# A Transformer-Based Machine Learning Framework for Risk Stratification of Left Bundle Branch Block After Transcatheter Aortic Valve Replacement

**DOI:** 10.3390/diagnostics16101422

**Published:** 2026-05-07

**Authors:** Hayoung Ahn, Sungwoo Hur, Cheol Hyun Lee, Se Hun Kang, Daeung Ohn, SookJung Kim, Junghoon Lee, Yeon-Jik Choi, Jeong-Eun Yi, Suk Min Seo, Sung-Won Jang, Won Hwa Kim, Osung Kwon

**Affiliations:** 1 Graduate School of AI, Pohang University of Science and Technology, Pohang 37673, Republic of Korea; ahnha@postech.ac.kr (H.A.); hursungwoo@postech.ac.kr (S.H.); 2 Division of Cardiology, Department of Internal Medicine, Keimyung University Dongsan Hospital, Deagu 42601, Republic of Korea; movicbeat@naver.com; 3 Department of Cardiology, CHA Bundang Medical Center, CHA University, Seongnam 13488, Republic of Korea; kirara00@gmail.com; 4 Division of Cardiology, Department of Internal Medicine, Eunpyeong St. Mary’s Hospital, College of Medicine, The Catholic University of Korea, Seoul 03312, Republic of Korea; ondae@naver.com (D.O.); lovelysj0426@gmail.com (S.K.); me@leejunghoon.com (J.L.); lozenge0602@gmail.com (Y.-J.C.); jung30134@naver.com (J.-E.Y.); ssm530@catholic.ac.kr (S.M.S.); clement@naver.com (S.-W.J.); 5 Computer Science and Engineering, Pohang University of Science and Technology, Pohang 37673, Republic of Korea; 6 Institut Cardiovasculaire Paris-Sud, Hôpital Privé Jacques Cartier, Ramsay-Santé, 91300 Massy, France

**Keywords:** machine-learning, left bundle branch block, transcatheter aortic valve replacement, risk stratification

## Abstract

**Background/Objectives**: Left bundle branch block (LBBB) remains a common complication after transcatheter aortic valve replacement (TAVR) and is associated with adverse clinical outcomes. However, accurate prediction of LBBB remains challenging due to the complex interactions among the anatomical, procedural, and clinical factors. This study aimed to develop a machine learning (ML)-based framework to predict LBBB and identify relevant contributing features. **Methods**: In this multicenter retrospective study, we analyzed 242 patients undergoing TAVR across three institutions. A machine learning framework incorporating transformer-based feature selection and conventional classifiers was developed. Model performance was evaluated using accuracy, precision, recall, F1-score, and area under the receiver operating characteristic curve (AUC). Internal validation was performed using bootstrap resampling. **Results**: The gradient boosting model using ML-derived features demonstrated the most balanced performance, achieving an accuracy of 78.05% and an F1-score of 50.46%, with modest discrimination (AUC 0.61). The ML-based approach identified clinically relevant features, including coronary height, left ventricular outflow tract/annulus ratio, and prosthetic valve size, as well as additional variables not emphasized in conventional analyses. **Conclusions**: ML-based feature selection can capture complex feature interactions beyond traditional statistical approaches and provide clinically meaningful insights into risk stratification for LBBB after TAVR. Although predictive performance was modest, this approach highlights the potential of ML for improved risk stratification and individualized procedural planning. Further large-scale external validation is warranted.

## 1. Introduction

Transcatheter aortic valve replacement (TAVR) has become an established treatment for severe aortic stenosis across the full spectrum of surgical risk and is increasingly performed in younger and lower-risk populations [1]. Despite substantial improvements in device technology and procedural techniques, leading to high procedural success rates and favorable clinical outcomes, left bundle branch block (LBBB) remains a frequent complication, with reported incidences ranging from 4% to 65% [2,3,4]. LBBB reflects a conduction system injury, resulting in delayed electrical activation of the left ventricle, which may impair coordinated cardiac contraction. Development of LBBB after TAVR is associated with adverse outcomes, including atrioventricular block, increased need for permanent pacemaker implantation, and impaired long-term cardiac function [5,6,7].

Although multiple anatomical, procedural, and electrocardiographic predictors have been proposed, the incidence of LBBB has not significantly decreased over time [3,4]. This persistent risk highlights the complex and multifactorial nature of conduction disturbances after TAVR. Conventional statistical approaches may be limited in capturing the interactions among the diverse clinical and imaging variables that contribute to LBBB development.

Given the expanding indication of TAVR in lower-risk patients, accurate pre-procedural risk stratification for new-onset persistent (NOP) LBBB—defined as LBBB persisting until discharge—is increasingly important for optimizing procedural planning and patient management [7]. In this context, the machine learning (ML) approaches offer an opportunity to explore complex relationships within the heterogeneous datasets and may provide complementary insights beyond the traditional analytical methods. Therefore, the present study aimed to develop an ML-based framework integrating clinical and imaging data to evaluate the factors associated with NOP-LBBB after TAVR and to explore whether ML-driven feature selection could provide additional insights for risk stratification.

## 2. Methods

### 2.1. Database and Study Population

**Figure 1:**
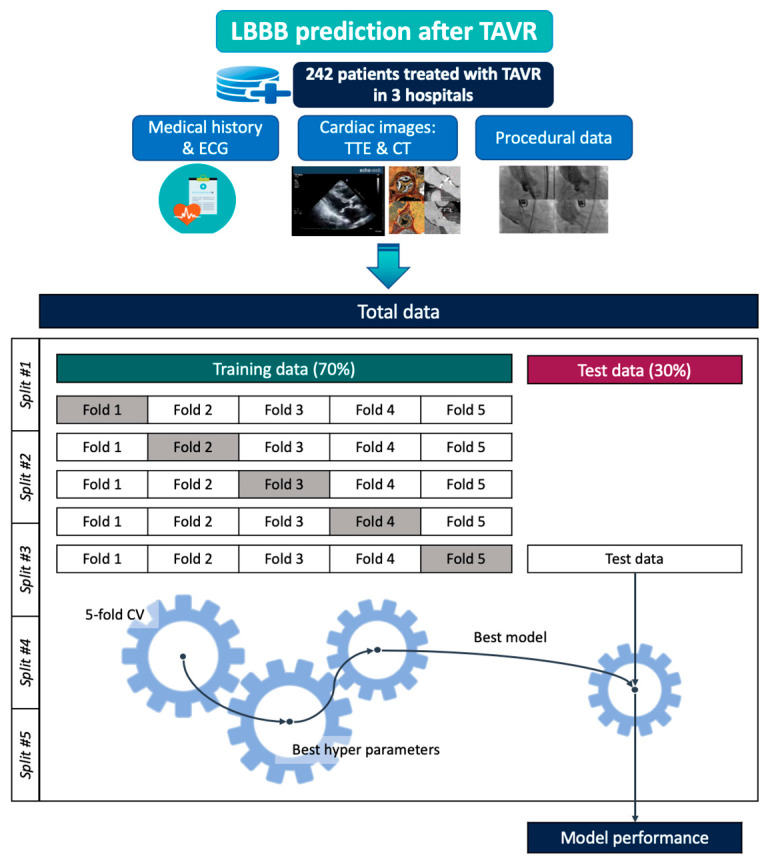
Study diagram. Various clinical data from the 242 TAVR patients collected across three institutions were combined and divided into training (70%) and test (30%) sets for model development and evaluation. The nested validation framework consisted of five bootstrap resampling iterations (outer loop), each using a different fixed random seed to create 70/30 train–test splits, with an inner 5-fold cross-validation within the training set to optimize hyperparameters and evaluate model performance. CT, computed tomography; CV, cross-validation; ECG, electrocardiography; LBBB, left bundle branch block; TAVR, transcatheter aortic valve replacement; TTE; transthoracic echocardiography.

From 2018 to 2022, a total of 242 patients who underwent TAVR across three institutions were included in this study, after excluding those with baseline LBBB or pre-existing pacemaker implantation (Eunpyeong St. Mary hospital: 152 patients; Bundang CHA Hospital: 38 patients; Keimyung Dongsan Hospital: 52 patients) (Figure 1). We defined NOP-LBBB as any new LBBB occurring during the hospitalization period after the TAVR procedure that persisted until hospital discharge, including the patients who died during the hospitalization without proven resolution of the LBBB [7]. The details of the variables in each category are presented in Table 1 and Supplemental Table S1. Data collection and preparation were approved by the institutional review boards of each center, including Eunpyeong St. Mary’s Hospital, and the requirement for informed consent was waived.

### 2.2. Model Architecture

**Figure 2:**
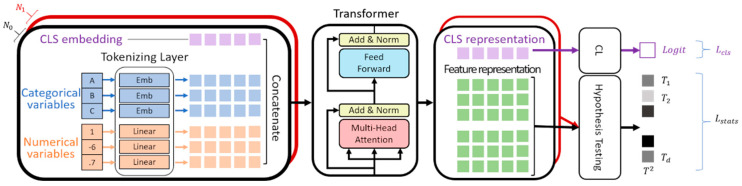
Overall model architecture. The model processes clinical and imaging variables to learn relationships among features and identify those associated with NOP-LBBB. Input variables are transformed into feature representations and refined by transformer layers to capture the interactions among the variables. A hypothesis testing layer evaluates feature importance, while a classification layer generates the final prediction for LBBB. The model is trained using two complementary objectives: a classification loss ($L_{cls}$) to optimize the prediction performance, and a statistical loss ($L_{stat}$) to enhance the separation between patients with and without NOP-LBBB, thereby supporting the feature selection. Additionally, the black box represents a set of $N_{0}$ negative LBBB samples, while the red box corresponds to a set of $N_{1}$ positive LBBB samples. CL, classification layer.

**Figure 3:**
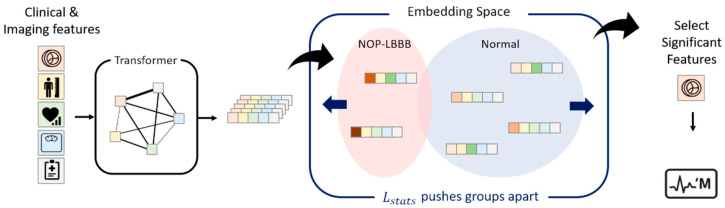
Schematic diagram illustrating feature representation learning and selection. Input features undergo initial tokenization and embedding. These initial representations are processed by the transformer blocks, which utilize the attention mechanisms to generate the contextually enriched feature representations. A core component of the model’s training objective is driven by the statistical loss ($L_{stat}$), which aims to maximize the Hotelling’s *T*^2^ statistic computed from these representations, thereby promoting the separability between the LBBB and non-LBBB patient groups. Following training, the statistical significance of each feature is determined by calculating the *T*^2^ statistic and its associated *p*-value based on the final learned representations, resulting in the “OURS” feature set. Independently, an aggregated representation (CLS) is passed to a classification head to generate the final LBBB risk prediction. NOP-LBBB, new-onset persistent left bundle branch block.

Our framework is designed to identify which clinical and imaging features are most strongly associated with NOP-LBBB by learning about the complex relationships among the variables and selecting the most informative ones. As shown in Figure 2, our method consists of three main modules: tokenizing layers, transformer layers, and a hypothesis testing and classification layer (CL). The embedding and transformer layers are used to extract meaningful feature representations for feature selection, and their design follows the standard transformer architecture [8]. The CL is defined as a fully connected output layer that receives the embedding of the classification token (CLS) as input and generates the prediction logit for the LBBB classification task. In this framework, the transformer captures interactions among features, and the Hotelling’s *T*^2^ test identifies those that best distinguish between patients with and without NOP-LBBB (Supplemental Figure S1). This process is implemented through a hypothesis-testing layer that applies Hotelling’s $T^{2}$ test to the learned feature representations, while CL stabilizes model training through classification regularization. A comprehensive description of the proposed architecture, along with a schematic overview of the entire workflow, is presented in Figure 3. A detailed description of the model architecture, including mathematical formulation and implementation details, is provided in the Supplementary Methods.

### 2.3. Experimental Design

Baseline Methods and Evaluation Metric. To evaluate the feature selection capability of our method, we conducted a comparative performance analysis using the same dataset with three different feature sets: the first feature set, referred to as the ALL set, includes all available features; the second set, referred to as the DK set, consists of features selected based on domain knowledge; and the third feature set, referred to as the OURS set, contains features selected by our method. For each feature set, we searched for an optimal classification model among logistic regression, decision tree, random forest, support vector machine, linear discriminant analysis, quadratic discriminant analysis, and gradient boosting and evaluated the quality of a feature set by comparing the best LBBB classification model that each set recorded. This assessment was conducted by comparing key metrics, including accuracy, recall, precision, and F1-score, to determine the effectiveness of a feature set. Additionally, model performance was further examined using receiver operating characteristic (ROC) curves and calibration plots.

Although our model was trained using a classification objective, the primary goal of this framework was to identify the informative features rather than to optimize the classification performance. Therefore, instead of directly evaluating the model outputs, we assessed performance by training separate classifiers using the features selected by OUR method. This approach allows a more objective evaluation of the selected features. According to the No Free Lunch Theorem [9], no single model can universally outperform all others across all possible datasets. The effectiveness of a model is inherently dependent on the characteristics of the given dataset. Accordingly, we evaluated multiple models to identify the most suitable approach for our task.

Training-validation-test data split. We employed five repeated 70/30 train–test resampling iterations, which enables more efficient utilization of the available data and provides more stable performance estimates by reducing variance. Specifically, we collected data from three different hospitals and performed five bootstrap resampling iterations (Figure 1). The proposed method was evaluated by dividing the dataset into training and test sets at a 7:3 ratio across five fixed random seeds. For each random seed, models were trained on the entire training dataset using hyperparameters optimized through a 5-fold cross-validation and subsequently assessed on the previously separated test data.

Hyperparameters. For the transformer, we used a single transformer layer with 8 heads and set the dimension of the tokenizing layers to 32. As for the optimization, we set both coefficients of loss functions: $\lambda_{1}$ and $\lambda_{2}$ as $1\times 10^{-5}$, and optimized total loss using the Adam optimizer with a learning rate of $1\times 10^{-4}$, while keeping all the other settings at their default values in the PyTorch [10]. In addition, we optimized our method for 3500 epochs using full-batch training. To validate the robustness of these settings, we additionally performed a sensitivity analysis on the key hyperparameters, the results of which are summarized in Supplemental Table S2.

Clinical analysis vs. OURS. To evaluate the consistency of our method’s feature selection with the existing medical knowledge, we compared the feature set identified by our method with the well-established significant clinical features. A confusion matrix was reported to highlight the agreement between the two feature sets. Additionally, to enhance model interpretability, we applied the Shapley additive explanations (SHAP) analysis to the final model trained on the OURS feature set.

All analyses were implemented in Python (version 3.10) using the PyTorch (version 1.13.1) and scikit-learn libraries (version 1.3.2). The preprocessing pipeline involved schema-based data type conversion, categorical label encoding, and numerical standardization to ensure data consistency. Subsequently, statistical feature selection was performed using transformer-derived feature embeddings, and significant features were identified based on Hotelling’s *T*^2^ test. The full implementation code and training scripts are available at our GitHub repository: https://github.com/ahnha-ahnha/lbbb-ft-main (accessed on 3 November 2025) [11].

## 3. Results

### 3.1. Baseline Characteristics and Event Rates

The baseline characteristics of the population are listed in Table 1 and Supplemental Table S1. The mean patient age was 82.6 ± 5.8 years; of the 242 patients, 86 (35%) were male. The STS-PROM score was 6.3 ± 5.2%, and the EURO score II was 5.7 ± 9.1%. A normal sinus rhythm was observed in 209 (86.4%) patients. NOP-LBBB occurred within the first day after TAVR in 51 (21%) patients. The QRS interval was significantly shorter in the NOP-LBBB group (95.6 ± 17.5 ms) compared to the no NOP-LBBB group (102.3 ± 20.2 ms, *p* = 0.03). A self-expanding valve was used more frequently in the NOP-LBBB group (54.9%) than in the no NOP-LBBB group (33.5%) (*p* = 0.01). Annular area (401.7 ± 71.2 mm^2^ vs. 435.3 ± 87.3 mm^2^, *p* = 0.01), annular perimeter diameter (23.0 ± 2.0 mm vs. 23.9 ± 2.3 mm, *p* = 0.01), and left ventricular outflow tract area (380.2 ± 86.2 mm^2^ vs. 430.8 ± 105.8 mm^2^, *p* = 0.002) were significantly smaller in the NOP-LBBB group. In addition, the left ventricular outflow tract area annular ratio was lower in the NOP-LBBB group (0.9 ± 0.1) than in the no NOP-LBBB group (1.0 ± 0.1, *p* = 0.01). Total calcium volume was significantly smaller in the NOP-LBBB group (354.4 ± 295.1 mm^3^) than in the no NOP-LBBB group (507.6 ± 553.4 mm^3^, *p* = 0.01).

### 3.2. LBBB Classification Results

Table 2 presents the accuracy, recall, precision, and F1-score of the logistic regression, decision tree, random forest, support vector machine, linear discriminant analysis, quadratic discriminant analysis, and gradient boosting across three distinct feature selection settings: ALL, DK, and OURS. In the ALL dataset, the support vector machine achieved the highest accuracy at 79.27 ± 0.03%. However, its precision, recall, and F1-score were relatively low, measuring 11.11 ± 0.11%, 50.00 ± 0.50%, and 18.18 ± 0.18%, respectively. In the DK dataset, the gradient boosting algorithm attained the highest accuracy of 76.83 ± 0.04%, while also demonstrating relatively balanced precision, recall, and F1-scores of 47.78 ± 0.08%, 50.00 ± 0.06%, and 48.83 ± 0.07%, respectively. In the OURS dataset, the gradient boosting algorithm achieved the highest accuracy of 78.05 ± 0.05% along with the most-balanced precision, recall, and F1-scores, measuring 47.78 ± 0.08%, 53.47 ± 0.09%, and 50.46 ± 0.08%, respectively.

As shown in Supplemental Figure S2, the ROC curves indicate that the OURS model achieved a greater area under the ROC curve (0.61) compared with the ALL (0.46) and DK (0.53) models, suggesting an overall improvement in the discrimination performance. The calibration plot of the gradient boosting model using the selected features (OURS) showed an overall agreement between the predicted probabilities and the observed event rates. In addition, based on the ROC analysis, the optimal clinical decision threshold was determined using Youden’s J statistic, which identified a threshold of 0.10 as the optimal cut-off probability. At this threshold, the model achieved a sensitivity of 63%, a specificity of 54%, and an overall accuracy of 58%, indicating its potential utility for identifying the patients at high risk of developing NOP-LBBB after TAVR, although the overall performance remained modest.

### 3.3. Clinical vs. Prediction Analysis

**Figure 4:**
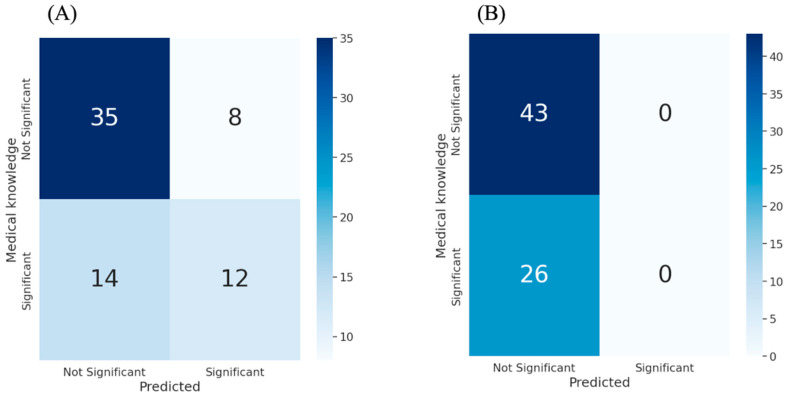
Confusion matrices comparing feature selection approaches. (**A**) Confusion matrix comparing features identified by medical knowledge and those selected by the OURS method. (**B**) Confusion matrix comparing features identified by medical knowledge and those selected by logistic regression. The Y-axis of matrices represents clinically significant features, determined based on medical knowledge, while the X-axis represents the model’s predicted significant features. The significance level (α) was set at 5% in both cases. LR, logistic regression.

In this experiment, we selected 26 features as clinically established LBBB-related features based on the clinical observations and evidence from the clinical literature [12,13]. The confusion matrix illustrates the alignment of our method with medical knowledge in identifying significant features. The results show that our method correctly identified 12 significant features while correctly classifying 35 features as irrelevant (Figure 4A). Assuming medical knowledge as the ground truth, this result demonstrate the strong capability of our method in identifying significant features (accuracy = 68.11%, precision = 60.00%, recall = 46.15%, and F1-score = 52.17%). Furthermore, it is particularly noteworthy as traditional methods such as logistic regression fail to identify any significant feature at a 5% significance level (Figure 4B). In contrast, our method is trained to recognize such features without prior medical knowledge.

In addition, we compared the significant features identified by our method with those determined based on established medical knowledge in Supplemental Table S3. Several risk factors recognized in traditional clinical knowledge align with those discovered using the OURS method. These include the weight, left ventricular ejection fraction, aortic valve area measured by echocardiography, left posterior fascicular block, first-degree atrioventricular block, PR interval on electrocardiography, prosthetic valve generation, prosthetic valve size, bicuspid valve, annular mean diameter, annular perimeter-derived diameter, and the left ventricular outflow tract/annulus ratio assessed by computed tomography (CT) scan. Meanwhile, variables such as height, peripheral vascular disease, the Society of Thoracic Surgeons Predicted Risk of Mortality (STS-PROM) score, sinotubular junction area, diameter of the right coronary cusp, and left and right coronary heights measured by CT scan were identified exclusively by the OURS method. In the SHAP analysis (Supplemental Figure S3), right coronary height emerged as the most influential feature, followed by the left ventricular outflow tract/annulus ratio, left coronary height, prosthetic valve size, right coronary cusp diameter, annular mean diameter, and PR interval. The SHAP summary plot illustrated the overall direction and magnitude of each feature’s contribution to the model predictions.

## 4. Discussion

In this multicenter study, we applied a machine learning (ML)–based framework to evaluate factors associated with NOP-LBBB after TAVR. The main findings can be summarized as follows: (1) the gradient boosting model demonstrated modest but balanced predictive performance; (2) ML-based feature selection identified clinically relevant variables associated with NOP-LBBB; and (3) several additional features not consistently emphasized in conventional analyses were identified, particularly from CT–derived parameters.

Approximately 30% of patients develop LBBB immediately after TAVR, with around 15% persisting at discharge [3,4]. NOP-LBBB is clinically relevant, as it is associated with an increased risk of atrioventricular block, permanent pacemaker implantation, and adverse cardiac remodeling [5,6,7,14,15]. Although its impact on mortality remains debated, several studies, including a recent meta-analysis, have reported an association with increased all-cause mortality [7,16,17,18]. Numerous studies have attempted to identify predictors of conduction disturbances after TAVR. Pre-procedural factors include baseline conduction abnormalities, calcification of the aortic valve or left ventricular outflow tract, prolonged QRS duration, prior coronary artery bypass grafting, and female sex [19,20,21]. Procedural factors such as membranous septum length, implantation depth, valve oversizing, and the use of self-expanding devices have also been implicated [19,20,21,22,23].

Despite recognizing these risk factors and accounting for them during the procedure, the incidence of LBBB has remained largely unchanged, underscoring the need to better understand the complex and multifactorial mechanisms underlying conduction disturbances after TAVR. Conventional analytical approaches may be limited in capturing interactions among diverse clinical, anatomical, and procedural variables. In this context, ML provides an opportunity to explore such multidimensional relationships and to uncover the patterns that may not be readily apparent using traditional methods.

In the present study, the ML-based framework demonstrated modest but balanced predictive performance. Notably, this performance was achieved without relying on predefined domain knowledge, highlighting the ability of the model to autonomously identify informative features from heterogeneous data. This domain knowledge–independent approach is particularly relevant in complex clinical settings such as TAVR, where interactions between anatomy, device characteristics, and procedural strategies are not fully understood. Importantly, beyond predictive performance, the model consistently identified clinically relevant features, supporting its potential role in feature discovery and hypothesis generation. However, the overall performance of the evaluated models, reflected in a maximum F1-score of 50.46%, suggests that the dataset may have lacked a sufficient sample size to adequately represent the underlying data distribution. Additionally, the limited dataset size may have constrained the models’ ability to generalize effectively, potentially lacking the informative variance needed to achieve higher predictive power. Factors such as class imbalance, feature noise, and a mismatch between model complexity and dataset characteristics may also have contributed to the suboptimal predictive performance. Taken together, these findings suggest that the current model may have limited applicability in direct clinical decision-making. Further studies with larger sample sizes and more advanced modeling strategies, including additional feature engineering and ensemble approaches, are warranted to improve predictive performance and clinical utility.

While there was considerable overlap with traditionally recognized risk factors for NOP-LBBB, the ML-based approach identified additional variables that provide further insight into the underlying mechanisms of conduction disturbances after TAVR. In particular, CT-derived parameters such as coronary height emerged as important contributors. Coronary height has traditionally been considered primarily in the context of coronary obstruction risk. However, our findings suggest that it may also be indirectly associated with conduction disturbances. Coronary height may influence procedural decisions, particularly implantation depth, which can in turn affect the risk of conduction system injury. In patients with relatively low coronary height, operators may adopt deeper valve implantation to mitigate the risk of coronary obstruction. Given that deeper implantation is a well-established risk factor for conduction system injury, this may partly explain the observed association between coronary height and NOP-LBBB. In addition to anatomical factors, clinical variables such as peripheral vascular disease, prior myocardial infarction, and STS-PROM score were also identified. Advanced atherosclerosis, conduction system defects resulting from prior myocardial infarction, and overall risk assessed by the STS-PROM score may collectively indicate the fragility of the cardiac conduction system. This may explain why conduction disturbances occur even in the absence of clearly identifiable procedural risk factors. Overall, these findings suggest that both anatomical and clinical factors may interact to influence the risk of conduction disturbances.

From a clinical standpoint, these findings have several potential implications. Pre-procedural identification of high-risk patients using readily available clinical and imaging parameters may support more tailored procedural strategies. For example, the careful consideration of implantation depth in patients with low coronary height, as well as the avoidance of excessive prosthesis oversizing in vulnerable patients or advanced atherosclerotic patients, may help reduce the risk of post-procedural conduction disturbances. These findings may therefore provide complementary information to assist procedural planning, although they are not yet sufficient to guide clinical decision-making independently. Further validation is warranted to confirm the reproducibility of these findings across different populations and procedural settings.

### Limitations

Several limitations of this study should be acknowledged. First, this was a retrospective analysis of a relatively small multicenter cohort, which may limit the representativeness of the findings. Although the inclusion of multiple centers may improve generalizability, the sample size may not fully capture the heterogeneity of the broader TAVR population. Second, and most importantly, an independent external validation cohort was not available, which represents a major limitation of this study and may affect the generalizability of the model. To address this, we employed a multicenter dataset along with repeated bootstrap resampling and cross-validation to enhance the robustness of model evaluation. Nevertheless, these approaches cannot substitute for true external validation, and the results should therefore be interpreted with caution. We are currently expanding our collaboration with additional institutions and plan to conduct a dedicated prospective external validation in future work. Third, the complex and multifactorial nature of NOP-LBBB after TAVR, combined with potential class imbalance, may have inherently limited the predictive performance of the model. Finally, although we incorporated CT-derived parameters, direct integration of imaging data, such as raw CT or echocardiographic information, was not performed and may further improve model performance in future studies.

## 5. Conclusions

In this multicenter study, an ML–based framework demonstrated modest but consistent predictive performance for new-onset persistent LBBB after TAVR and identified clinically relevant factors associated with this complication. These findings were obtained without relying on predefined domain knowledge and suggest that ML may be useful for exploring the interactions among clinical and anatomical variables and for supporting risk stratification. However, given the relatively small sample size and the absence of external validation, these results should be interpreted with caution. Further prospective multicenter studies with external validation are warranted to confirm these findings and to establish their clinical applicability.

## Figures and Tables

**Table 1:** Clinical characteristics, baseline echocardiography, and electrocardiographic parameters of the study population, according to the occurrence of new-onset persistent left bundle branch block.

	Total Population (N = 242)	No NOP-LBBB (N = 191)	NOP-LBBB (N = 51)	*p* Value
**Clinical Characteristics**				
Age (year)	82.57 ± 5.8	82.3 ± 6.0	83.6 ± 4.9	0.14
Male	156 (35.5)	118 (61.8)	38 (74.5)	0.13
BMI (kg/m^2^)	23.9 ± 4.0	23.9 ± 4.0	23.5 ± 3.9	0.50
Hypertension	187 (77.3)	144 (75.4)	43 (84.3)	0.25
Diabetes mellitus	88 (36.4)	71 (37.2)	17 (33.3)	0.73
Hyperlipidemia	101 (41.7)	83 (43.5)	18 (35.3)	0.37
NYHA classification				0.98
I-II	39 (16.1)	31 (16.2)	8 (15.7)	
III-IV	203 (83.9)	160 (83.8)	43 (84.3)	
Chronic lung disease	25 (10.3)	21 (11.0)	4 (7.8)	0.69
eGFR < 60 mL/min/1.73 m^2^	92 (38.0)	72 (37.7)	20 (39.2)	0.97
Coronary artery disease	125 (51.7)	105 (55.0)	20 (39.2)	0.07
Previous ischemic stroke	25 (10.3)	20 (10.5)	5 (9.8)	0.98
Peripheral vascular disease	12 (5.0)	10 (5.2)	2 (3.9)	0.98
Previous valve surgery	4 (1.7)	3 (1.6)	1 (2.0)	0.99
Previous history of MI	10 (4.1)	7 (3.7)	3 (5.9)	0.76
Previous history of PCI	88 (36.4)	70 (36.6)	13 (25.5)	0.19
Previous history of CABG	2 (0.8)	2 (1.0)	0 (0.0)	0.99
STS-PROM score (%)	6.3 ± 5.2	6.2 ± 5.0	6.5 ± 5.8	0.67
EURO score I (%)	14.8 ± 14.9	13.9 ± 14.5	17.7 ± 16.0	0.17
EURO score II (%)	5.7 ± 9.1	5.9 ± 9.7	4.9 ± 6.5	0.45
**Baseline Echocardiography**				
LVEF (%)	56.4 ± 13.1	55.9 ± 13.5	57.9 ± 11.4	0.34
LVEF < 50%	53 (21.9)	45 (23.6)	8 (15.7)	0.31
Aortic valve Vmax (m/s)	4.4 ± 0.8	4.4 ± 0.8	4.1 ± 0.7	0.01
Aortic valve mean gradient (mmHg)	45.4 ± 17.4	47.1 ± 17.9	39.3 ± 13.9	0.001
Aortic valve area (cm^2^)	0.8 ± 0.2	0.7 ± 0.2	0.7 ± 0.2	0.59
Aortic regurgitation, moderate-severe	119 (49.2)	97 (50.8)	22 (43.1)	0.42
**Baseline Electrocardiography**				
Normal sinus rhythm	209 (86.4)	166 (86.9)	43 (84.3)	0.80
Atrial fibrillation/flutter	35 (14.4)	27 (14.1)	8 (15.7)	0.97
Left anterior fascicular block	7 (2.9)	7 (3.7)	0 (0.0)	0.36
Left posterior fascicular block	1 (0.4)	1 (0.5)	0 (0.0)	0.99
1st degree AV block	15 (6.2)	12 (6.3)	3 (5.9)	0.99
2nd degree AV block	2 (0.8)	1 (0.5)	1 (2.0)	0.89
PR interval (ms)	166.9 ± 57.8	165.3 ± 57.3	173.3 ± 59.6	0.39
QRS interval (ms)	100.9 ± 19.8	102.3 ± 20.2	95.6 ± 17.5	0.03
Corrected QT interval (ms)	458.1 ± 35.6	458.3 ± 35.5	457.3 ± 36.2	0.85

Data are the mean ± standard deviation or numbers (%). Abbreviations: AV, atrioventricular; BMI, body mass index; CABG, coronary artery bypass graft; eGFR, estimated glomerular filtration rate; LBBB, left bundle branch block; LVEF, left ventricular ejection fraction; NOP, new-onset persistent; MI, myocardial infarction; NYHA, New York Heart Association; PCI, percutaneous coronary intervention; STS-PROM, Society of Thoracic Surgeons Predicted Risk of Mortality.

**Table 2:** Performance of left bundle branch block classification.

Model	Method	Accuracy (%)	Recall (%)	Precision (%)	F1-Score (%)
Logistic Regression	ALL	74.39 ± 0.02	11.11 ± 0.11	25.0 ± 0.25	15.38 ± 0.15
	DK	63.41 ± 0.00	26.67 ± 0.07	23.61 ± 0.07	24.81 ± 0.04
	OURS	60.98 ± 0.02	26.67 ± 0.07	21.83 ± 0.00	23.57 ± 0.03
Decision Tree	ALL	56.10 ± 0.07	16.11 ± 0.06	13.39 ± 0.01	13.88 ± 0.02
	DK	48.78 ± 0.02	16.11 ± 0.06	10.05 ± 0.02	12.24 ± 0.03
	OURS	56.10 ± 0.02	11.11 ± 0.11	8.33 ± 0.08	9.52 ± 0.10
Random Forest	ALL	76.83 ± 0.01	0.00 ± 0.00	0.00 ± 0.00	0.00 ± 0.00
	DK	68.29 ± 0.02	0.00 ± 0.00	0.00 ± 0.00	0.00 ± 0.00
	OURS	75.61 ± 0.00	5.56 ± 0.06	16.67 ± 0.17	8.33 ± 0.08
Support Vector Machine	ALL	79.27 ± 0.03	11.11 ± 0.11	50.00 ± 0.50	18.18 ± 0.18
	DK	63.41 ± 0.00	10.56 ± 0.01	13.39 ± 0.01	11.76 ± 0.00
	OURS	62.20 ± 0.01	10.56 ± 0.01	12.70 ± 0.02	11.44 ± 0.00
Linear Discriminant Analysis	ALL	76.83 ± 0.01	0.00 ± 0.00	0.0 ± 0.00	0.00 ± 0.00
	DK	52.44 ± 0.01	21.67 ± 0.12	13.37 ± 0.04	16.30 ± 0.07
	OURS	54.88 ± 0.04	32.78 ± 0.23	18.06 ± 0.10	23.06 ± 0.14
Quadratic Discriminant Analysis	ALL	78.05 ± 0.02	15.56 ± 0.04	75.00 ± 0.25	24.29 ± 0.04
	DK	67.07 ± 0.04	20.56 ± 0.09	25.00 ± 0.13	22.55 ± 0.11
	OURS	75.61 ± 0.02	16.11 ± 0.06	41.67 ± 0.08	23.08 ± 0.08
**Gradient Boosting**	ALL	74.39 ± 0.01	5.00 ± 0.05	16.67 ± 0.17	7.69 ± 0.08
	DK	76.83 ± 0.04	47.78 ± 0.08	50.00 ± 0.06	48.83 ± 0.07
	**OURS**	**78.05 ± 0.05**	**47.78 ± 0.08**	**53.47 ± 0.09**	**50.46 ± 0.08**

The ALL setting utilizes all available features from the dataset, while DK includes features selected based on domain knowledge. In contrast, OURS trains each model using features selected by our method. Values are presented as mean ± standard deviation across five repeated 70/30 hold-out evaluations, and the best-performing score is highlighted in bold.

## Data Availability

The data that support the findings of this study are available from the corresponding author upon reasonable request. The code used in this study is publicly available at: [https://github.com/ahnha-ahnha/lbbb-ft-main] (accessed on 3 November 2025).

## Supplementary Materials

The following supporting information can be downloaded at: https://www.mdpi.com/article/10.3390/diagnostics16101422/s1, Supplementary Methods: Detailed Description of the Machine Learning Framework. Table S1. Procedural and computed tomography parameters of the study population, according to the occurrence of new-onset persistent left bundle branch block. Table S2. Sensitivity analysis of transformer hyperparameters on feature selection count and average classification performance. Table S3. Comparison of significant features selected by OURS and those identified through medical knowledge. Figure S1. α-level Hotelling’s *T*^2^ test. Figure S2. Receiver operating characteristic and calibration plots for model performance evaluation. Figure S3. Shapley additive explanations analysis of the baseline gradient boosting model for predicting new-onset persistent left bundle branch block.

## Abbreviations

The following abbreviations are used in this manuscript:

CT	computed tomography
CL	classification layer
CLS	classification token
LBBB	left bundle branch block
ML	machine learning
NOP	new-onset persistent
ROC	receiver operating characteristic
SHAP	Shapley additive explanations
STS-PROM	Society of Thoracic Surgeons predicted risk of mortality
TAVR	transcatheter aortic valve replacement
