# Supplementary material for: A Transformer-Based Machine Learning Framework for Risk Stratification of Left Bundle Branch Block After Transcatheter Aortic Valve Replacement
Source: Diagnostics (Basel). 2026 May 7;16(10):1422. doi: 10.3390/diagnostics16101422 (PMC13206223; doi:10.3390/diagnostics16101422)

Supplemental Materials

H Ahn et al. "A Transformer-Based Machine Learning Framework for Risk Stratification of Left Bundle Branch Block After Transcatheter Aortic Valve Replacement"

Supplementary Methods: Detailed Description of the Machine Learning Framework

1) Notation.

2) Tokenizing layers.

3) Transformer layers.

4) Classification layer and hypothesis testing layer.

5) Objective function

Supplementary Table S1. Procedural and computed tomography parameters of the study population, according to the occurrence of new-onset persistent left bundle branch block.

Supplementary Table S2. Sensitivity analysis of transformer hyperparameters on feature selection count and average classification performance.

Supplementary Table S3. Comparison of significant features selected by OURS and those identified through medical knowledge.

Supplementary Figure S1. α-level Hotelling's T^2 test.

Supplementary Figure S2. Receiver operating characteristic and calibration plots for model performance evaluation.

Supplementary Figure S3. Shapley additive explanations analysis of the baseline gradient

boosting model for predicting new-onset persistent left bundle branch block.

This supplementary material was provided by the authors to provide readers with additional information of their work.

Supplementary Methods: Detailed Description of the Machine Learning Framework

1) Notation. We first define the following notations to explain our method effectively. Let $S = \{(x_i, , y_i)\}_{i=1}^{N}$ be a dataset of N observations, where x_i is an observation and y_i denotes its corresponding label. Each $x_i = (x_i^{num}, x_i^{cat}) \in \mathbb{R}^{d^{num}} \times \mathbb{R}^{d^{cat}}$ consists of a concatenated vector of numerical and categorial features, and x_{ij}^{num} and x_{ij}^{cat} represent numerical and categorical features, respectively, and thus the total number of features is $d = d^{num} + d^{cat}$. For LBBB classification $y_i \in \{0,1\}^N$ and the cardinality of the dataset S is $N = N_0 + N_1$, where N_0 and N_1 are the number of 0-labeled and 1-labeled samples, respectively. In this work, we label positive LBBB samples as 1.

2) Tokenizing layers. The tokenizing layers project numeric and categorical features into a latent space with the same dimension. Specifically, D-dimensional embeddings of numerical features e_{ij}^{num} and categorical features e_{ij}^{cat} are computed as follows:

$$e_{ij}^{num} = b_j^{num} + x_{ij}^{num} W_j^{num},$$

$$e_{ij}^{cat} = x_{ij}^{cat} W_j^{cat},$$

where $b_j^{num} \in R$ and $W_j^{num} \in \mathbb{R}^D$ are parameters of a linear projection layer of jth numerical feature, and $W_j^{cat} \in \mathbb{R}^{C_j \times D}$ is a lookup table (embedding matrix) of jth categorial feature, where C_j is the number of classes within j^{th} feature. The final embedding of an observation x_i, e_i is the concatenation of e^{cls}, $[e_{i1}^{num}; e_{i2}^{num}; e_{i3}^{num}; \dots; e_{id^{num}}^{num}]$ and $[e_{i1}^{cat}; e_{i2}^{cat}; e_{i3}^{cat}; \dots; e_{id^{cat}}^{cat}]$, where e^{cls} is an embedding vector of [CLS] token which is later used for classification task, and ; denotes concatenation of vectors.

3) Transformer layers. The transformer layers take the final embedding e_i compute the followings to obtain feature representations rich in semantics.

1. Linear Projections: For each head h, the input e_i is projected into three different spaces to create query, key, and value vectors.

$$Q_h = e_i W_h^Q, \qquad \mathrm{K_h} = e_i W_h^K, \qquad V_h = e_i W_h^V$$

where W_h^Q, W_h^K, $W_h^V \in R^{D \times D_k}$ are learnable projection matrices.

2. Scaled Dot-Product Attention: For each head h, the attention weights are computed by taking the dot product of the query Q_h with all keys K_h, scaling by $\sqrt{D_k}$, and applying the softmax function:

$$Attention_h(Q_h, K_h, V_h) = softmax\left(\frac{Q_h^T K_h}{\sqrt{D_k}}\right)\mathrm{V_h}.$$

3. Concatenation of Heads: The outputs of the different attention heads are concatenated and then projected by another learnable weight matrix $W^O \in \mathrm{R}^{HD_k \times D}$, where H is the number of attention heads.

$$MultiHead(e_i) = \bigoplus_{h=1}^{H} Attention_h(Q_h, K_h, V_h)\, W^O.$$

We denote the final output of $MultiHead(e_i)$ as $z_i \in R^{d \times D}$. Note that $z_i[0]$, the first-row vector of z_i is the [CLS] representation, and the remaining rows, $z_i[1:]$ correspond to feature representations.

4) Classification layer and hypothesis testing layer. The final layer consists of two fundamental layers, CL and hypothesis testing layer. As illustrated in the **Figure 2**, CL takes [CLS] representation as an input and outputs the logits for classification. Specifically, we

used Layer Normalization followed by ReLU activation and a simple linear layer, as follows [1]:

$$ReLU\left(LayerNorm\left(e_i^{cls}\right)\right)W^{out},$$

where $ReLU(v) = max(v, 0)$ for an arbitrary vector v, and $W^{out} \in \mathbb{R}^D$.

The hypothesis testing layer computes Hotelling's T^2 statistics using $Z[1:]$ (Feature representations stacked in a matrix). The intuition behind this idea is that Hotelling's T^2 statistic can be naturally applied to define a group separation loss for vector representations, which simultaneously maximizes inter-cluster variance while minimizing intra-cluster variance [2]. For two j^{th} mean feature representations from 1-labeled and 0-labeled samples, $\bar{z}_j{}^{(1)}$ *and* $\bar{z}_j{}^{(0)}$ (respectively), and for two j^{th} covariance matrices from the two different samples, $\Sigma_j^{(1)}$ and $\Sigma_j^{(0)}$, the Hotelling's T^2 statistics of jth feature between the two groups, T_j is formally computed as follows:

$$T_j = \Delta_j^T \Sigma_j^{pooled} \Delta_j,$$

where $\Delta_j^T = \bar{z}_j{}^{(1)} - \bar{z}_j{}^{(0)}$ is the mean difference vector between the two groups, and Σ_j^{pooled} is the pooled covariance matrix that is computed as:

$$\Sigma_j^{pooled} = \frac{(N_1 - 1)\Sigma_j^{(1)} + (N_0 - 1)\Sigma_j^{(0)}}{N_1 + N_0 - 2}.$$

In addition, to obtain p-values from our method, F_j, the F-statistics of jth feature representations, is calculated as:

$$F_j = \frac{(N_1 + N_0 - p - 1)}{(p \times (N_1 + N_0 - 2))} \times \mathrm{T_{j,}}$$

and the corresponding p-value is obtained as:

$$p - value_j = 1 - P(F \leq F_j),$$

where $F \sim F(p, N_0 + N_1 - p - 1)$. Finally, as shown in **Figure 3**, we can use this $p-values$ to obtain significant features under a certain significant level, α. i.e., the jth feature is significant if $p - value_j, \leq \alpha$.

5) Objective function

To learn meaningful feature representations and identify significant features, we propose minimizing the inverse logarithm of the sum of T_j across all group comparisons (thus, maximizing the T^2 statistic) while regularizing it with a classification task and the l_2 norm of the model parameters $l_2(\Theta)$. This loss L is formally written as:

$$L = L_{stat} + \lambda_1 l_2(\Theta) + \lambda_2 CrossEntropy(y, \hat{y})$$

$$= \frac{1}{\log\left(\Sigma_{j=1}^{d} T_j + 1\right)} + \lambda_1 \|\Theta\|_2^2 - \lambda_2 \frac{\Sigma_{i=1}^{N} \log P_\Theta(y_i|x_i)}{N}$$

where Θ is a set of model parameters, L_{stat} denotes the first term of L, $\frac{1}{\log\left(\Sigma_{j=1}^{d} T_j+1\right)}$, and the term +1 added to the $\Sigma_{j=1}^{d} T_j$ ensures numerical stability by preventing issues with zero values when computing the logarithm, $\Sigma_{i=1}^{N} \log P_\Theta(y_i|x_i) / N$ corresponds to the Cross Entropy loss between y and predicted label $\hat{y}$ (i.e., $CrossEntropy(y, \hat{y})$). Additionally, λ_1 and λ_2 are hyperparameters that control the strength of regularization and classification task.

Supplementary Table S1. Procedural and computed tomography parameters of the study population, according to the occurrence of new-onset persistent left bundle branch block.

**Procedural variables**				
	**Total population**	**No NOP-LBBB**	**NOP-LBBB**	***P* value**
Prosthetic valve type				0.01
Balloon-expandable	150 (62.0)	127 (66.5)	23 (45.1)	
Self-expanding	92 (38.0)	64 (33.5)	28 (54.9)	
New generation valve (Sapien 3/Ultra or Evolut R/Pro)	188 (77.7)	155 (81.2)	33 (64.7)	0.02
Prosthetic valve size				0.74
20	11 (4.5)	10 (5.2)	1 (2.0)	
23	83 (34.3)	64 (33.5)	19 (37.3)	
26	82 (33.9)	65 (34.0)	17 (33.3)	
29	63 (26.0)	49 (25.7)	14 (27.5)	
34	3 (1.2)	3 (1.6)	0 (0.0)	
Prosthetic valve oversizing	12.0 ± 7.0	11.5 ± 6.9	13.9 ± 7.4	0.05
Balloon pre-dilatation	84 (34.7)	73 (38.2)	11 (21.6)	0.04
Balloon post-dilatation	19 (7.9)	16 (8.4)	3 (5.9)	0.77
**Computed tomography parameters**				
Bicuspid valve	12 (5.0)	10 (5.2)	2 (3.9)	0.98
Annular short diameter (mm)	20.6 ± 2.2	20.9 ± 2.2	19.7 ± 2.0	0.001
Annular long diameter (mm)	26.4 ± 2.7	26.6 ± 2.7	25.6 ± 2.7	0.02
Annular mean diameter (mm)	23.5 ± 2.3	23.7 ± 2.4	22.8 ± 2.1	0.01
Annular area (mm^2)	428.1 ± 85.1	435.3 ± 87.3	401.7 ± 71.2	0.01

Annular area-derived diameter (mm)	23.3 ± 2.2	23.5 ± 2.3	22.5 ± 2.0	0.01
Annular perimeter (mm)	74.6 ± 7.3	75.2 ± 7.5	72.4 ± 6.3	0.02
Annular perimeter diameter (mm)	23.8 ± 2.3	23.9 ± 2.3	23.0 ± 2.0	0.01
SOV area (mm^2)	776.1 ± 152.7	779.0 ± 151.8	767.6 ± 157.0	0.67
Sinus annular ratio	4.6 ± 3.6	5.5 ± 4.1	1.9 ± 0.3	0.33
Diameter of NCC (mm)	32.6 ± 13.5	32.9 ± 15.2	31.4 ± 3.5	0.22
Diameter of LCC (mm)	31.2 ± 3.5	31.4 ± 3.5	30.8 ± 3.3	0.29
Diameter of RCC (mm)	30.3 ± 3.5	30.5 ± 3.6	29.6 ± 3.0	0.12
STJ area (mm^2)	638.0 ± 126.3	641.5 ± 126.9	627.6 ± 125.2	0.53
STJ annular ratio	1.5 ± 0.3	1.5 ± 0.3	1.6 ± 0.4	0.10
STJ mean diameter (mm)	28.0 ± 3.3	28.1 ± 3.4	27.6 ± 2.9	0.34
LCC-STJ height (mm)	20.9 ± 2.8	21.1 ± 2.8	20.2 ± 2.5	0.05
LVOT area (mm^2)	419.93 ± 103.85	430.8 ± 105.8	380.2 ± 86.2	0.002
LVOT annular ratio	1.0 ± 0.1	1.0 ± 0.1	0.9 ± 0.1	0.01
LVOT short diameter (mm)	20.0 ± 2.9	20.3 ± 2.9	18.8 ± 2.6	0.001
LVOT long diameter (mm)	26.8 ± 3.1	27.0 ± 3.2	26.0 ± 2.7	0.04
Total calcium volume (mm^3)	474.6 ± 512.2	507.6 ± 553.4	354.4 ± 295.1	0.01
LCC calcium volume (mm^3)	126.8 ± 153.8	141.3 ± 167.0	74.1 ± 70.2	<.001
RCC calcium volume (mm^3)	130.0 ± 179.7	140.9 ± 194.8	90.5 ± 100.8	0.02
NCC calcium volume (mm^3)	221.1 ± 242.3	230.4 ± 259.2	187.5 ± 165.2	0.17
Left coronary height (mm)	13.8 ± 2.8	13.4 ± 3.0	15.3 ± 2.6	0.52
Right coronary height (mm)	16.7 ± 2.6	16.7 ± 2.6	16.6 ± 2.9	0.75

Data are the mean ± standard deviation or numbers (%).

Abbreviations: LBBB, left bundle branch block; LCC, left coronary cusp; LVOT, left ventricular outflow tract; NCC, non-coronary cusp; NOP, new-onset persistent; RCC, right coronary cusp; SOV, sinuses of Valsalva; STJ, sinotubular junction.

Supplementary Table S2. Sensitivity analysis of transformer hyperparameters on feature selection count and average classification performance.

This table summarizes the results of the sensitivity analysis where the embedding dimension, number of transformer layers, and number of attention heads of the transformer were varied. For each configuration, the number of features selected by the OURS method ($p < 0.05$) and the average performance (accuracy and macro F1-score) across seven classifiers using the selected features are reported. As the results were comparable across configurations, the baseline configuration (Dimension = 32, Depth = 1, Heads = 8) was retained for consistency and interpretability.

Configuration	Depth	Heads	Dimension	Number of Selected Features	Average accuracy (%)	Average F1-score (%)
Baseline	1	8	32	20	66.20 ± 9.92	21.35 ± 14.53
Depth = 2 (vs baseline = 1)	2	8	32	40	69.35 ± 7.09	24.10 ± 20.41
Dimension = 16 (vs baseline = 32)	1	8	16	24	66.31 ± 9.91	20.91 ± 10.87
Heads = 16 (vs baseline = 8)	1	16	32	21	67.63 ± 3.70	22.53 ± 21.24

Supplementary Table S3. Comparison of significant features selected by OURS and those identified through medical knowledge.

Significant features of medical domain knowledge (DK) and OURS are highlighted in yellow and green colors, respectively.

Features	DK	OURS	*P* value for OURS	Features	DK	OURS	*P* value for OURS
**Clinical characteristics**				**Procedural variables**			
Age			0.25	Balloon pre-dilatation			0.10
Gender			0.52	Prosthetic valve type			0.25
Height			0.02	New generation valve (Sapien 3/Ultra or Evolut R/Pro)			0.001
Weight			0.04	Prosthetic valve size			0.02
Hypertension			0.23	Prosthetic valve over sizing			0.09
Diabetes mellitus			0.08	Balloon post-dilatation			0.54
Hyperlipidemia			0.13	**CT analysis**			
NYHA classification			0.24	Bicuspid valve			0.02
Chronic lung disease			0.75	Annular short diameter			0.13
eGFR: mL/min/1.73m^2			0.26	Annular long diameter			0.59
Coronary artery disease			0.20	Annular mean diameter			0.03
Previous ischemic stroke			0.08	Annular area			0.45
Peripheral vascular			0.01	Annular area-derived diameter			0.60

disease							
Previous valve surgery			0.23	Annular perimeter			0.11
Previous history of MI			0.03	Annular perimeter-derived diameter			0.02
Previous history of PCI			0.16	SOV area			0.05
Previous history of CABG			0.47	Sinus annular ratio			0.21
STS-PROM score			0.03	Diameter of NCC			0.20
EURO score I			0.70	Diameter of LCC			0.30
EURO score II			0.29	Diameter of RCC			0.01
**Baseline echocardiography**				STJ area			0.003
LVEF			0.02	STJ annular ratio			0.07
Aortic valve Vmax			0.08	STJ mean diameter			0.46
Aortic valve mean gradient			0.26	LCC-STJ height			0.09
Aortic valve area			0.02	LVOT area			0.06
Aortic regurgitation grade			0.40	LVOT annular ratio			0.003
**Baseline Electrocardiography**				LVOT short diameter			0.57
Normal sinus rhythm			0.09	LVOT long diameter			0.07
Left anterior fascicular block			0.34	Total calcium volume			0.08

Left posterior fascicular block			0.02	LCC calcium volume			0.18
Atrial fibrillation			0.59	RCC calcium volume			0.55
Atrial flutter			0.28	NCC calcium volume			0.33
1st degree AV block			0.01	Left coronary height			0.02
2nd degree AV block			0.42	Right coronary height			0.04
PR interval			0.01				
QRS interval			0.11				

Abbreviations: AV, atrioventricular; BMI, body mass index; CABG, coronary artery bypass graft; CT, computed tomography; eGFR, estimated glomerular filtration rate; LCC, left coronary cusp; LVEF, left ventricular ejection fraction; LVOT, left ventricular outflow tract; MI, myocardial infarction; NCC, non-coronary cusp; NYHA, New York Heart Association; PCI, percutaneous coronary intervention; RCC, right coronary cusp; SOV, sinuses of Valsalva; STS-PROM, Society of Thoracic Surgeons Predicted Risk of Mortality; STJ, sinotubular junction.

Supplementary Figure S1. α-level Hotelling's T^2 test.

In this step, significant features were determined by applying the Hotelling's T^2 test to the statistical representations derived from the transformer layers, as illustrated in Figure 2.

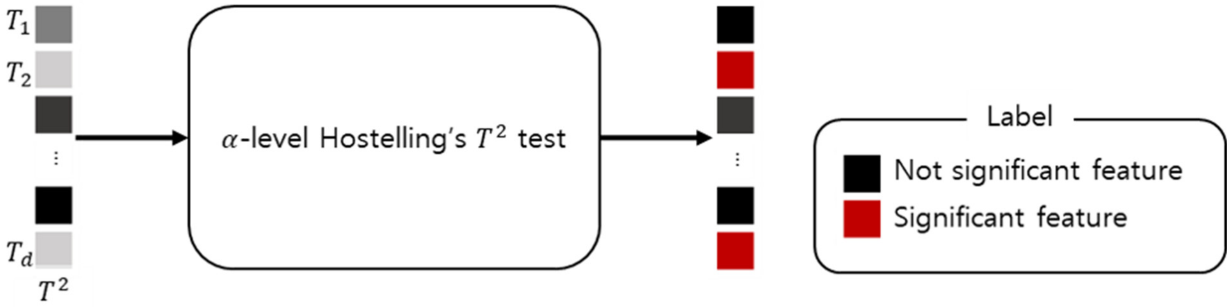

Supplementary Figure S2. Receiver operating characteristic and calibration plots for model performance evaluation. AUC, area under the curve.

(A) Receiver operating characteristic curves of the gradient boosting models comparing ALL, DK, and OURS approaches.

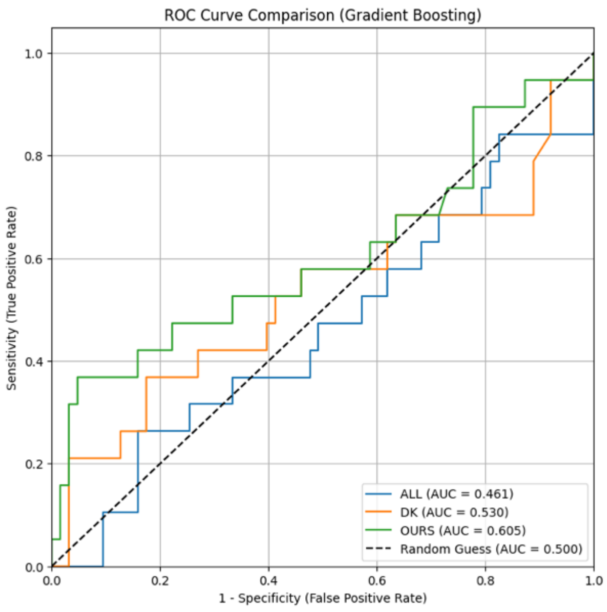

(B) Calibration plot of the gradient boosting model using the selected features (OURS).

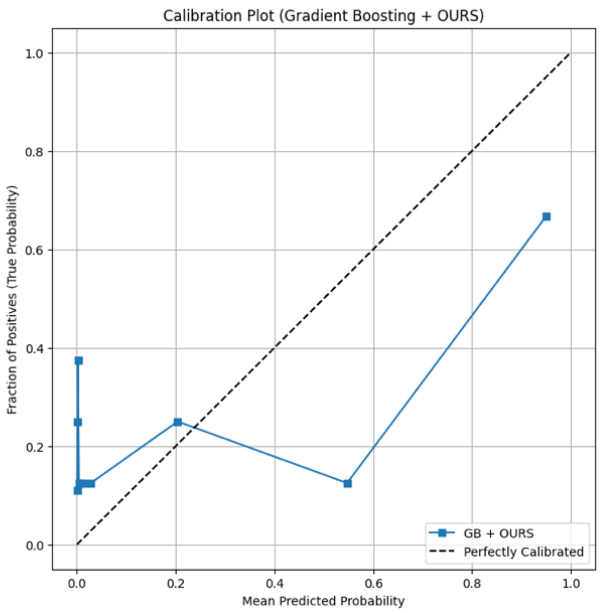

Supplementary Figure S3. Shapley additive explanations analysis of the baseline gradient boosting model for predicting new-onset persistent left bundle branch block.

LVEF, left ventricular ejection fraction; LVOT, left ventricular outflow tract; MI, myocardial infarction; RCC, right coronary cusp; STJ, sinotubular junction; STS-PROM, Society of Thoracic Surgeons Predicted Risk of Mortality.

(A) Global feature importance

Features were ranked by the mean absolute Shapley additive explanations (SHAP) value. Right coronary height was identified as the most influential feature, followed by LVOT/annulus ratio, left coronary height, prosthetic valve size, right coronary cusp diameter, annular mean diameter, and PR interval.

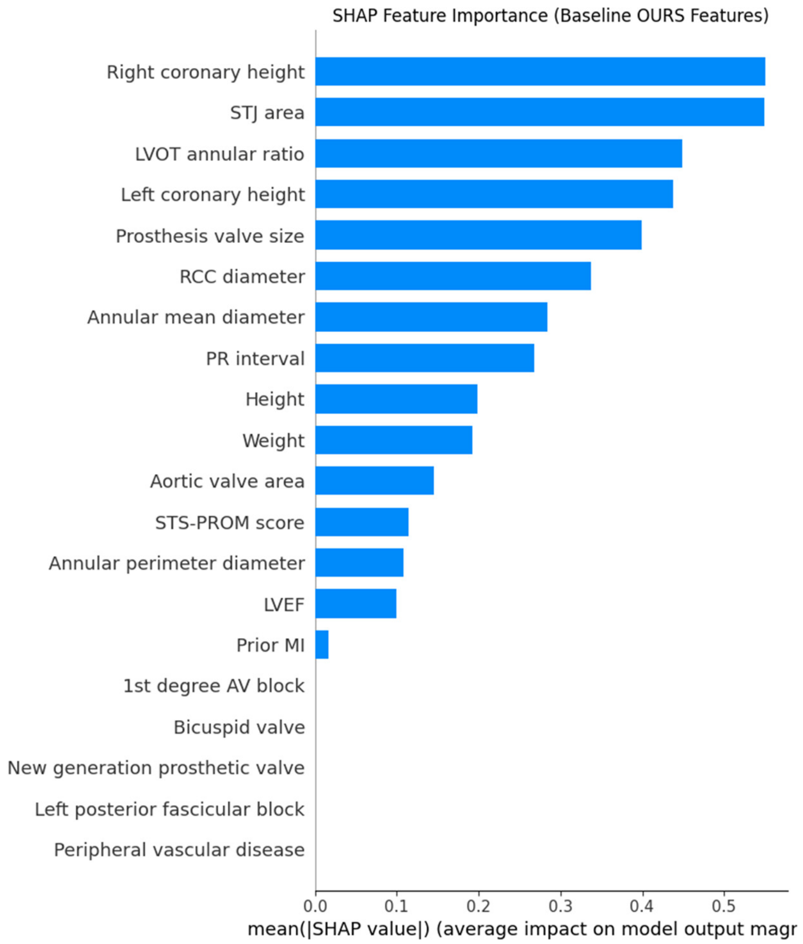

(B) Feature impact

The SHAP summary plot shows the direction and magnitude of each feature's contribution to individual predictions. Each point represents one patient, with SHAP values on the horizontal axis (positive values indicate increased LBBB risk) and color representing the feature value (red = high, blue = low). Lower coronary heights and smaller LVOT/annulus ratios (blue points) are associated with higher SHAP values, indicating that shorter coronary heights and narrower outflow tracts increase the predicted LBBB risk. Similarly, smaller right coronary cusp and annular diameters contribute to higher risk, while larger prosthetic valve size and longer PR intervals (red points) are also linked to increased LBBB likelihood.

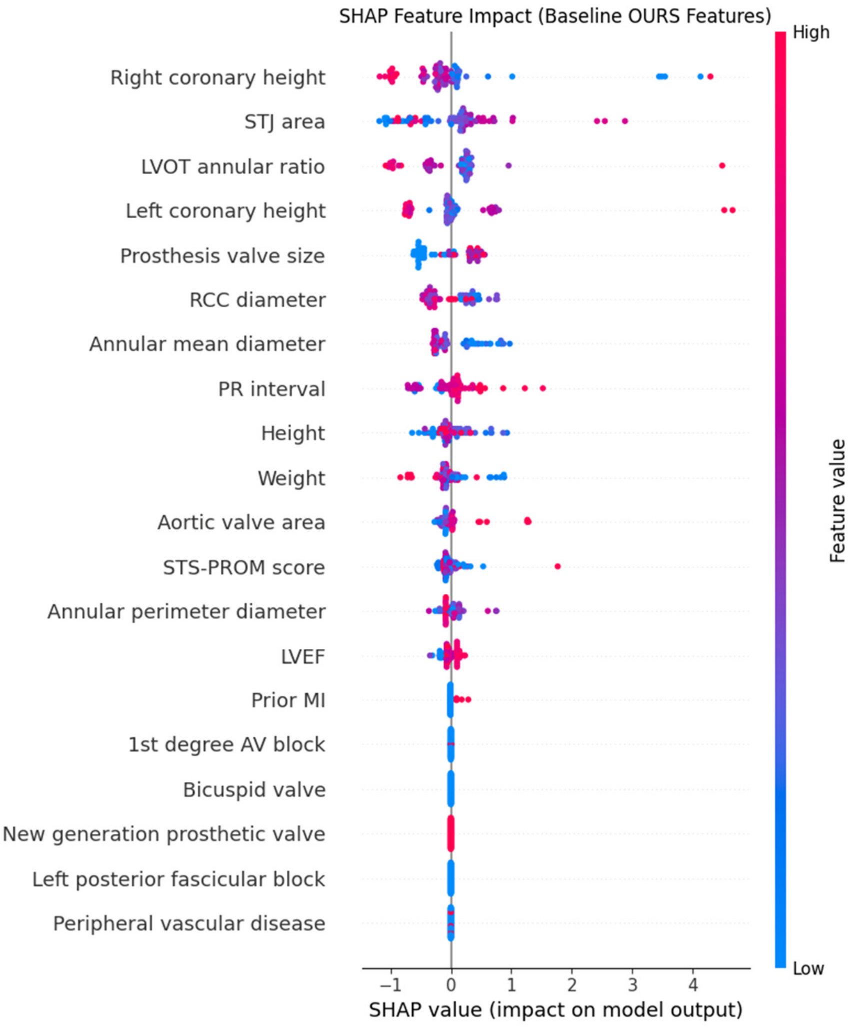

Supplement: Supplementary file 1 [file diagnostics-16-01422-s001.zip › diagnostics-4256761-supplementary/diagnostics-4256761-supplementary.pdf]
